# The effectiveness of educational intervention based on the Health Action Model (HAM) in improving breast cancer screening behaviors

**DOI:** 10.1186/s12905-023-02816-z

**Published:** 2024-01-03

**Authors:** Fahimeh Mahboobighazaani, Masoud Karimi, Mojtaba Azadbakht, Leila Ghahremani

**Affiliations:** 1 Student Research Committee, Department of Health Promotion, School of Health, Shiraz University of Medical Sciences, Razi Ave, Shiraz, Iran; 2 Qom University of Medical Sciences, Qom, Iran

**Keywords:** Breast cancer, Health action model, Screening behaviors, Breast self-examination, Clinical examination, Mammography

## Abstract

**Introduction:**

Breast cancer disease is known as the most common cancer among women. Lack of knowledge and awareness is a leading cause of breast cancer, and since nearly all women are increasingly susceptible to this disease, training screening behaviors for early detection is proven essential in order to reduce breast cancer mortality. Therefore, the present study was designed to determine the effect of educational intervention based on the Health Action Model in improving breast cancer screening behaviors in women aged 30 to 69 in Kashan, Iran.

**Methods:**

This quasi-experimental study was conducted on 162 women aged 30–69 years old among the clients of Comprehensive health service centers in Kashan, Iran and they were assigned to intervention and control groups. The research instrument included a questionnaire assessed within three phases: baseline, 3-months, and 6-months, containing Health Action Model (HAM) structures and also three screening behaviors. The intervention consisted of a model-based education package and was carried out over 2 months. To evaluate the effect of the intervention, the mean of model structures and proportion screening behaviors in the third and sixth months were compared with the baseline phase. All analyses were carried out using SPSS, version 22.

**Results:**

The intervention and control groups were homogeneous regarding the structures of the HAM and the proportion of screening behaviors in the baseline phase (*p* > 0.05). In the 3-month (*p* < 0.05) and 6-month (*p* < 0.05) phases, the mean scores of the HAM constructs in the intervention group were found higher compared with the control group. Moreover, the proportion of clinical breast examinations in the intervention group was statistically higher than in the control group in the 3-month (*p* < 0.001) and 6-month (*p* < 0.001) phases. In addition, the proportion of mammography performed in the 3-month (*p* = 0.002) and 6-month (*p* < 0.001) phases were reported to be higher in the intervention group compared with the control group.

**Conclusion:**

Overall, these results provide important insight into the effectiveness of the interventions based on the Health Action Model in promoting breast cancer screening behaviors and the determinants of such behaviors.

## Introduction

Breast cancer disease is one of the most common types of cancer in women and the second cause of death from cancer among women worldwide [1, 2]. More than 1.7 million new cases of breast cancer are known worldwide in 2021, accounting for about 25% of all cancers in women [3]. In the last few decades, an increased incidence of breast cancer has been grown rapidly diagnosing more than 40% of breast cancers in Asian countries [4]. It is estimated that 21.4% of all women’s cancers in Iran allocate to breast cancer [5]. It has been estimated that more than 2600 women die from this cancer yearly [6]. Early detection of cancer, followed by timely treatment, can be an effective control and preventive measure in which increases the chance of recovery and reduces the mortality, especially for high-risk groups population [7]. It appears that Screening may be considered an attainable approach in the early detection of breast cancer. It is an effective, simple, and affordable method to diagnose patients with precancerous lesions or early invasive cancers in the asymptomatic population [8].

Screening methods for early diagnosis included monthly breast self-examination, clinical examination by a midwife or physician and mammography [9]. Breast self-examination is an easy, effective and valuable method of breast cancer screening, which is suitable for all women and promotes their self-awareness [10]. Clinical breast examination is another screening method used by midwife or physician to diagnose lumps or other breast changes in women [11]. Additionally, mammography is the most sensitive and specific test which can be used for early diagnosis of breast cancer [12]. Due to the high cost of mammography and sometimes inaccessibility of the diagnostic test in healthcare centers in developing countries, including Iran, it seems that monthly breast self-examination as well as a subsequent examination by a midwife or physician could be an appropriate method in empowering women to diagnose breast cancer early in order to reduce breast cancer mortality [13, 14]. According to studies, only 3% to 17% of Iranian women regularly perform breast self-examination monthly compared to Western women [5, 15], also the rate of mammography is identified from 1.6 to 30.5 in Iran [16],while accurate information about the rate of clinical breast examination by midwife or physician has not been reported yet [5]. The studies have reported the following as the barriers to breast cancer screening for all women: lack of knowledge regarding breast cancer screening methods, lack of confidence in their ability to perform breast self-examination correctly, fear of finding a lump in the breast, embarrassment and shame caused by breast manipulation, absence of symptoms and concern due to the lack of awareness, lack of physicians’ recommendations, forgetting the breast self-examination schedule, pain caused by breast manipulation during the examination, the deficit in environmental support, cultural beliefs about fate, lack of support from spouse, friends, and families (social support), concerns about the high cost of mammography, pain during mammography, unpleasant test results, and lack of time [17–24]. Furthermore, lack of available information sources and expert personnel as well as the weakness of the referral systems make this problem stable [25].

Since several factors including personal, social and environmental factors are influential in performing breast cancer screening behaviors, using a comprehensive model with the set of influential factors seems necessary to carry out this intervention. If educational and interventional programs are performed based on the models and theories of health education, there would be more valuable and practical results. Hence, the research team decided to use the Health Action Model to conduct an educational intervention during the study after the literature review. The model was designed by Tones for the first time in the early 1970s. It incorporates the constructs of several models and theories selectively and practically and detects the psychological, social and environmental key factors that affect personal acceptance and actions related to health or illness [26]. The model consists of two main parts: 1) Systems that affect behavioral intention, such as belief, norms, motivation and self-concept systems and 2) Factors that affect belief, subjective norms, motivation and self-concept systems plus determining the possibility of converting behavioral intention into the performance such as skills, knowledge and environmental factors (physical, socioeconomic and sociocultural) [26]. Relatively a few studies have examined this model [27–30], but to our knowledge, there have been no attempts to study cancer screening behaviors regarding this model and there has been no comprehensive study carried out on three screening behaviors (including breast self-examination, examination by a midwife or physician and mammography). Therefore, the present study aimed to evaluate the effectiveness of an educational intervention based on the Health Action Model in order to improve breast cancer screening behaviors.

## Materials and methods

### Type of study and participants

The present quasi-experimental study was conducted from August 2021 to June 2022. The study took place at comprehensive healthcare centers in Kashan, Iran and the study population consisted of women aged 30 to 69 living in Kashan who had electronic health records in these centers. A sample size of 81 per group was obtained for the study using statistical software PASS (Power and Sample Size) version 15.05 which is a part of NCSS software [31] and a power of 0.95 and a drop of 15% (162 women in total).

### Sampling

For sampling, 8 comprehensive health service centers from four geographical regions of Kashan (2 centers from each regions) were selected. Then, from each center, the numbers of samples of that center were selected in proportion to the number of women aged 30–69 under the coverage of that center to the total sample size that met the inclusion criteria. Sampling in the centers was conducted through convenience sampling (available sampling) and it has been performing until we reached the desired sample size (162 women). During the sampling process, due to the covid-19 pandemy, people were not frequently attending the comprehensive health service centers and they just visited the centers to receive essential services like children vaccination or sometimes to receive midwifery services. Besides, it was not practically possible to use other sampling methods for the desired sample size; thus, the convenience sampling method was used. After reaching the desired sample size and obtaining informed and written consent from the participants, the samples were randomly (through lottery) divided into two intervention and control groups.

#### Inclusion criteria

The criteria for entering the study included: consent to participate in the study, women aged 30–69 years old, not suffering from breast, nervous and mental diseases, fifth grade education and above, not pregnant and breastfeeding, Iranian citizenship and having a smart phone.

#### Exclusion criteria

The exclusion criteria were: not being satisfied with the continuation of the study, leaving the virtual group (Whats App), not participating in the face-to-face educational program, migrating outside of Kashan and becoming pregnant during the study.

### Data collection tool

#### Measurements

The research instrument included a questionnaire in which after being designed and psychometrically performed, was used to collect information related to the performance of breast cancer screening behaviors by women and the factors affecting their performance [32]. The questionnaire contained 2 sections:

- **Section 1) Socio-demographic information questionnaire**
  Socio-demographic information containing variables: age, marital status, education level, occupation, insurance status, menopause status, income and the most important source of health information, were obtained through literature review and expert panel opinions.
- **Section 2) Health Action Model constructs questionnaire**
  The constructs of the Health Action Model containing variables: knowledge, perceived susceptibility, perceived severity, perceived barriers and benefits, perceived self-efficacy, motivation, subjective norms, self-concept, environmental factors, skill, behavioral intention and behavior, were obtained through literature review [33–41], semi-structured interviews, expert panel opinions and studies in this field.

The knowledge structure contained 12 items and the answers were ‘yes’, ‘no’ and ‘don’t know’. A correct answer was given a score of 1, and a no or I don’t know answer was given a score of 0. The score range was from 0 to 12.

The Health Action Model construct or behavior (breast cancer screening behaviors containing: monthly breast self-examination, clinical examination by a midwife or physician and mammography) with 6 items and scoring it as a Likert scale for item 2 and as yes and no for items 1, 3, 4, 5 and 6 were done. The score range was from 0 to 6.

For the constructs of the Health Action Model, except for knowledge and behavior, scoring was done on a 5-point Likert scale (strongly agree, agree, have no opinion, disagree and strongly disagree). A score of 5 was given to ‘completely agree’ answer and 1 to ‘completely disagree’ answer; Moreover, regarding the structure of perceived barriers, scoring was done in reverse. In this way, 5 points were given to ‘completely disagree’ answer and 1 point to ‘completely agree’ answer.

Perceived susceptibility structure with 3 items and score range from 3 to 15, perceived severity with 5 items and score range from 5 to 25, perceived barriers with 9 items and score range from 9 to 45, perceived benefits with 5 items and Score range from 5 to 25, perceived self-efficacy with 9 items and score range from 9 to 45, motivation with 4 items and score range from 4 to 20, subjective norms with 9 items and score range from 9 to 45, self-concept with 5 items and the score range was from 5 to 25, environmental factors with 4 items and score range from 4 to 20, skill with 10 items and score range from 10 to 50 and behavioral intention with 4 items and score range from 4 to 20.

Content validity was used to check the validity of the questionnaire. A panel consisting of 13 expert professors included health education and promotion, gerontology, the doctor in charge of the family health program of the deputy health department, experts from the family health department and experts of non-communicable diseases of Kashan health deputy investigated the validity of the content in qualitative and quantitative ways. The values of content validity ratio and content validity index for knowledge items, respectively (.93 & .94), for constructs of Health Action Model, respectively (.95 & .97) and for behavior items, respectively (.99 & .99) were obtained.

To check the construct validity of the constructs of the Health Action Model that had a Likert scale, confirmatory factor analysis was performed. After conducting confirmatory factor analysis, the questions with factors loading below 0.5 were excluded from the questionnaire.

Regarding the reliability of the tool, test-retest and Cornbrash’s alpha methods were used, and Cornbrash’s alpha value for knowledge and constructs of the health action model higher than 0.9 was obtained. To check the reliability of the behavior items, the Kappa coefficient of agreement was used, the values of which higher than 0.9 was obtained.

The questionnaire was completed by the target group over three periods of before, 3 and 6 months after the educational intervention. Before the intervention to check the status of performing screening behaviors in women and also the primary data analysis to prepare educational content, in the period of 3 and 6 months to check whether the educational intervention can be effective in performing screening behaviors or not.

#### Educational intervention

After selecting the samples and obtaining their informed and written consent, the interviewer attended the desired centers and completed the questionnaires by self-reporting in attendance interviews. After analyzing the results of the pre-test, the content and educational protocols were developed based on the most important predictors of breast cancer screening behaviors.

According to the social conditions in terms of the COVID-19 Pandemic, training of the intervention groups was done virtually through WhatsApp Messenger and face-to-face instruction for 2 months. An educational group titled “getting to know breast cancer” with 81 members was created in WhatsApp. The purpose of creating such educational group was explained to the participating women, then virtual education was carried out by sending educational videos and podcasts. Thus, the intervention group received training from the personnel of comprehensive health service centers as well as the compiled educational program. This educational program included six training sessions with videos and podcasts, the content of each one was made according to the constructs of the Health Action Model and reliable sources such as websites, books and other sources. The content in the educational podcast was the same as in the videos, which were prepared in audio form to remind the materials.

The educational content of videos and podcasts was based on the constructs of the health action model such as knowledge, perceived susceptibility (as feeling the risk of the breast cancer), perceived severity (feeling the seriousness of various complications, and physical, psychological, social consequences, and economic aspects), perceived benefits (perceiving the usefulness and application of breast cancer screening behaviors), perceived barriers (overcoming the barriers to performing breast cancer screening behaviors), self-efficacy (feeling confident in performing breast cancer screening behaviors), motivational factors, environmental and cultural factors, subjective norms, skills,behavioral intention and breast cancer screening behaviors.

After passing the severe course of the COVID-19 pandemic and virtual education ending, eight attendance training sessions (each session lasted for 2 h) were carried out by the researcher for the intervention group in the training hall of the Kashan health deputy (The intervention group was divided into two groups of 40 and 41 person and 4 sessions in which lasted 2 h were held for each group). The attendance education sessions included the use of Moulage educational aid as well as educational methods such as lecture, question and answer, group discussion, brainstorming, use of Moulage, slide shows and videos prepared in virtual education. Furthermore, the role-playing method was utilized to improve the skills and self-efficacy of women in performing breast self-examination. The intervention group was also continuously followed up by sending educational texts and voice messages. If they had any questions or problems, they asked in the WhatsApp educational group and received answers. The brainstorming method was also used on WhatsApp.

The women participating in the target group were advised to watch educational videos with other family members (i.e. influential people on breast cancer screening behaviors such as spouses, mothers, and sisters) to attract the support of influential people in line with the subjective norms construct. Only the control group received the routine training of comprehensive health service centers. The educational media used, the subject of education, the time spent for education, behavioral goals, the educational method and the structure used in education (virtual, face-to-face) are presented in Tables 1 and 2.

**Table 1:** Educational program presented in video clips and educational podcast

**Row**	Educational media	The subject of education	Time	Content expressed in the video clip	Behavioral goals	The structure used
1	Mobile and WhatsApp messenger	The importance of breast cancer	2ʹ, 59ʺ	The importance of breast cancer, the structure of the breast and the definition of breast cancer, presenting the incidence and mortality statistics of breast cancer in the country and the city of Kashan and the complications caused by it.	The audience will learn about breast cancer and its importance.	Knowledge, sensitivity and perceived intensity
2	Mobile and WhatsApp messenger	Causes of breast cancer	2ʹ, 7ʺ	Causes of cancer, breast cancer risk factors including behavioral and non-behavioral risk factors	The audience will get to know the behavioral and non-behavioral causes of breast cancer. The audience can state several behavioral/non-behavioral reasons. The audience should avoid these factors in their daily behaviour.	Knowledge, subjective norms, motivation for health action
3	Mobile and WhatsApp messenger	Breast cancer prevention and screening	2ʹ, 47ʺ	Prevention of breast cancer and performing healthy behaviors for prevention, the difference between early detection and screening, breast cancer screening methods for early detection	The audience should know the behaviors of breast cancer prevention. The audience should plan to perform these behaviors.	Knowledge, self-concept, self-efficacy
4	Mobile and WhatsApp messenger	Breast self-examination	4ʹ, 8ʺ	Breast examination by the person (self-examination) and how to do it, suspected signs and symptoms of breast cancer	The audience will find the skill of performing breast self-examination. The audience can perform breast self-examination and plan to do it monthly.	Self-efficacy, barriers and interests, skills, behavioral intention, behavior
5	Mobile and WhatsApp messenger	Clinical examination and mammography	3ʹ, 28ʺ	Clinical breast examination by midwife/doctor, mammography, ultrasound and MRI	The audience can overcome the obstacles of clinical examination / mammography. The audience should proceed to clinical examination/mammography. The audience can establish a proper relationship with the clinical examination / mammography agents.	Motivation, barriers, environmental factors, self-efficacy, behavioral intention, behavior
6	podcast	All the titles used in the video clips	15ʹ, 55ʺ	All content used in video clips	The audience should become familiar with breast cancer, its risk factors, ways of prevention and screening, how and when to perform screening behaviors, and plan to perform these behaviors.	Structures of health action model

**Table 2:** Educational program presented in face-to-face meeting

**Row**	learning assist tools and teaching methods	The subject of education	Time	Content expressed in the video clip	Behavioral goals	The structure used
1	TV - video projector – computer - PowerPoint –brain storming - questions and answer	The importance of breast cancer and the causes of breast cancer	2 h	The importance of breast cancer and the presentation of breast cancer incidence and mortality statistics in the country and the city of Kashan and the complications caused by it - breast cancer risk factors including behavioral and non-behavioral risk factors	The audience learn about breast cancer and its importance. The audience know the behavioral and non-behavioral causes of breast cancer. The audience can state several behavioral/non-behavioral reasons. The audience should avoid these factors in their daily behaviour.	Knowledge, perceived sensitivity and intensity, subjective norms, motivation
2	TV - video projector - computer - PowerPoint - brainstorming - question and answer - group discussion	Breast cancer prevention and screening	2 h	Prevention of breast cancer and performing healthy behaviors for prevention, the difference between early detection and screening, breast cancer screening methods for early detection	The audience should know the behaviors of breast cancer prevention. The audience should plan to perform these behaviors.	Knowledge, self-concept, self-efficacy
3	TV -video projector-computer-breast Molage -powerpoint- brain storming -question and answer-practical demonstration	Breast self-examination	2 h	Breast examination by the person (self-examination) and how to do it, suspected signs and symptoms of breast cancer	The audience will find the skill of performing breast self-examination. The audience can perform breast self-examination and plan to do it monthly.	Self-efficacy, barrier and benefits, skill, environmental factors, behavioral intention, behavior
4	TV - video projector - computer - PowerPoint - group discussion - questions and answer	Clinical examination and mammography	2 h	Clinical examination by a midwife/doctor, mammography and its time, ultrasound and MRI	The audience can overcome the obstacles of clinical examination / mammography. The audience should proceed to clinical examination/mammography. The audience can establish a proper relationship with the clinical examination / mammography agents.	Motivation, barriers, environmental factors, self-efficacy, behavioral intention, behavior

#### Data analysis

In order to describe the participants, descriptive statistics indicators (frequency, percentage, mean and standard deviation) were used. The normality of data distribution was checked with skewness and kurtosis indices, and since the values of these two indices for dependent variables were in the range of -2 to +2, parametric tests were used. Independent t-tests and chi-square tests were used to compare the mean and proportion in the intervention and control groups, respectively. In order to evaluate the effect of the intervention on the dependent variables, the difference between the mean/proportion of the dependent variables in the post-intervention phases (3-month and 6-month phase) and the baseline phase was separately calculated for both groups. Afterwards, the difference between the two groups (difference in differences) was compared, in which independent t-tests (comparison of the mean difference of two groups) and chi-square (comparison of the difference of behavior proportion in two groups) were used. The collected data were analyzed using SPSS (Statistical Package for the Social Sciences) 22. The significance level was considered *p* < 0.05.

#### Ethical considerations

The present study was approved by the Research Ethics committee of Shiraz University of Medical Sciences with the ethical code IR.SUMS.REC.1400.349. The informed consent forms were completed by the participants before starting the study and they were assured that the project results would remain confidential. Furthermore, the educational content of the intervention group was provided to the control group at the end of the educational intervention period.

## Results

**Fig. 1:**
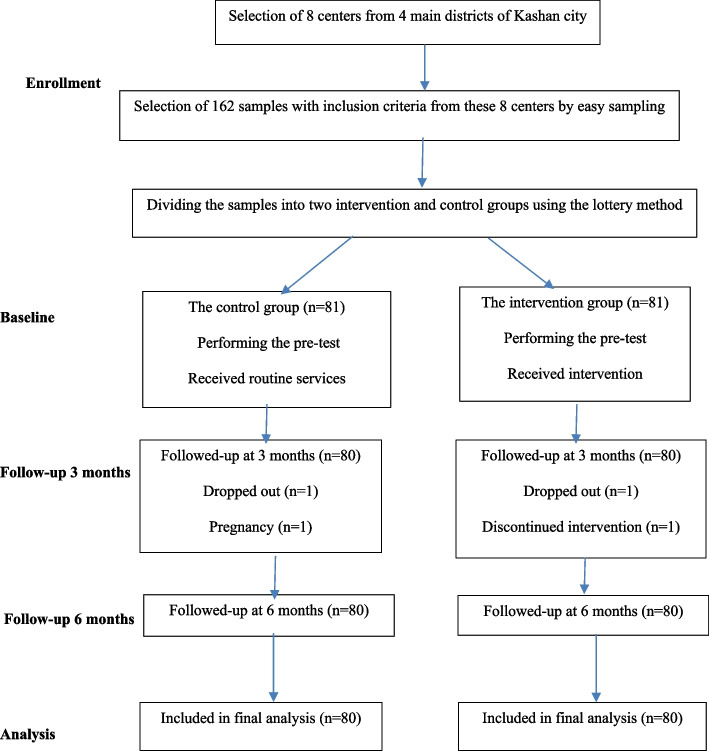
CONSORT diagram of participant flow

In this investigation, 162 women aged 30–69 years old in Kashan, Iran were studied to examine their breast cancer screening behaviors. Among them, one woman in the intervention group (due to non-cooperation with the continuation of the study according to numerous follow-ups) and one woman in the control group (due to pregnancy) were excluded from the study. Therefore, the analysis was performed on 160 women (Fig. 1). The mean ± standard deviation of the women’s age was 43.84 ± 9.46 and 42.21 ± 8.52 years old in the intervention and control groups, respectively. The mean ± standard deviation of the women’s number of pregnancies was 2.14 ± 1.51 and 2.28 ± 1.48 in the intervention and control groups with no significant difference. Table 3 provides other information about the demographic variables related to the intervention and control groups. There was a significant difference between the intervention and control groups in terms of job, but there was no significant difference in terms of education levels, marital status, insurance, menopausal status, and monthly income level.

**Table 3:** Comparison of statistical indices of demographic variables in the intervention and control groups

**Variable**	**Group intervention (80)** **(number/percent)**	**Group control (80)** **(number/percent)**	*P*-value*
Job			
Housewife	56 (44.1)	71 (55.9)	.003
Employee	24 (72.7)	9 (27.3)	
Education			
Diploma and under	52 (65)	52 (65)	1
Associate and upper	28 (35)	28 (35)	
Marital status			
Married	68 (85)	73 (91.3)	.676
Single	7 (8.8)	4 (5)	
Divorced	3 (3.8)	2 (2.5)	
Widow	29 (2.5)	1 (1.3)	
Insurance			
Have	75 (93.8)	79 (98.8)	.096
Does not have	5 (6.3)	1 (1.3)	
Menopause			
Yes	23 (28.8)	17 (21.3)	.273
No	57 (71.3)	63 (78.8)	
Monthly income			
Low	52 (44.8)	64 (55.2)	.073
Moderate	21 (67.7)	10 (32.3)	
Many	7 (53.8)	6 (46.2)	

Significant level (*p* < 0.05)

*P*-value *chi-square test

The inter-group comparison did not show any significant differences between the intervention and control groups in terms of the mean scores of the constructs of the health action model and breast self-examination behavior before the intervention; but a significant difference after the intervention was evident. The within-group comparison indicated a significant difference between the intervention and control groups in terms of the mean scores of the constructs of the Health Action Model in 3-month and 6-month phases; however, no significant difference between mean scores of perceived susceptibility, perceived barriers, perceived benefits, and behavioral intention in the control group in three time periods was found. Other constructs of the Health Action Model in this group revealed significant differences in the three periods after the intervention. In both the 3-month (*p* < 0.001) and 6-month (*p* < 0.001) phases, the average of breast cancer self-examination behaviors in the intervention group was significantly higher compared to the control group, while no significant difference was found at the baseline phase (*p* = 0.497) (Table 4).

**Table 4:** Comparison the constructs of the Health Action Model and breast self-examination behavior in the intervention and control groups

**The constructs of the** Health Action Model	**Groups**	Difference Between groups Mean ± SD			Mean difference within groups	
		baseline	3-month	6-month	Baseline to 3-month	Baseline to 6-month
	*P value					
**Knowledge**	intervention	8.36 ± 2.2	10.64 ± 1.5	11.57 ± 0.74	2.28	3.21
	control	8.15 ± 2.7	7.46 ± 2.7	6.89 ± 2.6	-0.69	-1.26
	*p*	0.598, t = 0.528	< 0.001, t = 8.878	< 0.001, t = 15.119	< 0.001, t = 8.582	< 0.001, t = 13.634
**Perceived susceptibility**	intervention	11.94 ± 2.4	13.42 ± 1.4	13.76 ± 1.2	1.49	1.82
	control	11.49 ± 2.6	11.46 ± 2.2	11.44 ± 1.8	0.02	-.05
	*p*	0.271, t = 1.106	< 0.001, t = 6.493	< 0.001, t = 9.240	< 0.001, t = 5.204	< 0.001, t = 5.951
**Perceived severity**	intervention	22.50 ± 2.7	23.38 ± 1.9	23.68 ± 1.5	.87	1.17
	control	21.75 ± 3.23	21.05 ± 3.15	20.71 ± 2.82	.7	-1.04
	*p*	0.119, t = 1.570	< 0.001, t = 5.607	< 0.001, t = 8.195	< 0.001, t = 3.964	< 0.001, t = 5.326
**Perceived barrier**	intervention	24.89 ± 6.1	19.95 ± 5.1	17.47 ± 3.7	-4.94	-7.41
	control	26.14 ± 7.0	26.55 ± 6.3	26.89 ± 6.0	.41	0.75
	*p*	0.231, t = -1.201	< 0.001, t = -7.254	< 0.001, t = -11.843	< 0.001, t = -6.371	< 0.001, t = -10.291
**Perceived benefits**	intervention	22.71 ± 2.7	23.39 ± 2.1	23.85 ± 1.6	.67	1.14
	control	22.13 ± 3.3	21.41 ± 3.3	21.35 ± 3.1	-.71	-.77
	*p*	0.221, t = 1.229	< 0.001, t = 4.398	< 0.001, t = 6.228	< 0.001, t = 3.353	< 0.001, t = 4.662
**Self-efficacy**	intervention	38.72 ± 5.1	40.81 ± 4.1	41.70 ± 3.3	2.09	2.97
	control	37.03 ± 6.0	36.06 ± 6.2	35.37 ± 5.9	-.96	-1.65
	*p*	0.058, t = 1.911	< 0.001, t = 5.710	< 0.001, t = 8.296	< 0.001, t = 4..266	< 0.001, t = 6.377
**Subjective norm**	intervention	37.40 ± 5.57	38.69 ± 4.83	39.78 ± 4.2	1.29	2.37
	control	36.43 ± 6.2	35.28 ± 5.74	34.13 ± 5.20	-1.14	-2.34
	*p*	0.360, t = .918	< 0.001, t = 3.889	< 0.001, t = 7.550	< 0.01, t = 3.169	< 0.001, t = 5.916
**Skill**	intervention	41.75 ± 5.6	44.55 ± 4.7	45.80 ± 4.0	2.80	4.05
	control	40.70 ± 5.92	39.83 ± 5.9	39.06 ± 5.4	-.87	-1.64
	*p*	0.251, t = 1.152	< 0.001, t = 5.551	< 0.001, t = 9.008	< 0.001, t = 4.651	< 0.001, t = 7.110
**Behavioral intention**	intervention	17.08 ± 2.6	18.16 ± 2.4	18.99 ± 1.7	1.08	1.91
	control	16.72 ± 2.6	16.35 ± 2.6	16.27 ± 2.5	-.37	-.45
	*p*	0.394, t = .854	< 0.001, t = 4.532	< 0.001, t = 8.180	< 0.001, t = 3.848	< 0.001, t = 6.623
**Breast self- exam**	intervention	1.73 ± 2.2	2.94 ± 1.4	3.51 ± 0.7	1.21	1.79
	control	1.99 ± 2.6	1.01 ± 1.4	0.76 ± 0.9	-.97	-1.22
	*p*	0.497, t = -.680	< 0.001, t = 7.359	< 0.001, t = 18.816	< 0.001, t = 6.448	< 0.001, t = 8.601

Significant level (*p* < 0. 01)

^*^P value t-test

At the baseline phase, the proportion of clinical breast examinations and mammography in both groups did not show a significant difference (*p* > 0.05). The proportion of clinical breast examinations in the intervention group was higher than in the control group in the 3-month (*p* < 0.001) and 6-month (*p* < 0.001) phases. In addition, the proportion of mammography performed in the 3-month (*p* = 0.002) and 6-month (*p* < 0.001) phases were reported to be higher in the intervention group than in the control group (Table 5).

**Table 5:** Proportion of performing clinical breast exam and mammography in the 3 phases of the study and comparing the 3- and 6-month phases with the baseline

**Screening behaviors**	**Groups**	Difference Between group N (%)			Chang within groups N (%)	
	*P value	Baseline	3-month	6-month	Baseline to 3-month	Baseline to 6-month
**Clinical breast exam**	intervention	9 (39.1)	45 (91.8)	76 (87.4)	36 (0.45)	67 (0.83)
	control	14 (60.9)	4 (8.2)	11 (2.6)	-10 (0.12)	-3 (0.03)
	*p*	0.260, X^2^ = 5.026	< 0.001, X^2^ = 49.450	< 0.001, X^2^ = 19.879	< 0.001, X^2^ = 1.269	< 0.001, X^2^ = 13.057
**Mammography**	intervention	3 (33.3)	9 (100)	24 (96)	6 (0.07)	21 (0.26)
	control	6 (66.7)	0 (0)	1 (4)	0	-5 (0.06)
	*p*	0.303, X^2^ = 1.860	< 0.01, X^2^ = 9.536	< 0.001, X^2^ = 13.611	< 0.001, X^2^ = 2.818	< 0.001, X^2^ = 6.632

Significant level (*p* < 0. 01)

*P*-value *chi-square test

## Discussion

The present study aimed to determine the effectiveness of an educational intervention based on the Health Action Model in improving breast cancer screening behaviors in women aged 30 to 69 in Kashan, Iran. In this study, demographic variables such as educational level, marital status, insurance status, menopause, and income levels were not significantly different in the two groups, but the intervention and control groups were significantly different in terms of the job.

The results of the present study indicated a significant difference between the intervention and control groups in terms of scores of knowledge 3 and 6 months after the intervention. The knowledge score of women in the intervention group increased significantly compared to the control group. Changes in individuals’ knowledge were consistent with the results of studies done by Bakhtariagdam et al. [42], Anwar Alameer et al. [43], and Sargazi et al. [44]. Various studies have identified that women have low to moderate knowledge about the symptoms, and risk factors of breast cancer. Thus, the benefits of breast cancer screening, and improving the level of knowledge and public attitude can have a positive role in performing breast cancer screening behaviors [45–47].

The findings clearly indicated that the perceived susceptibility score increased significantly in the intervention group after the educational intervention based on the health action model. Our results were consistent with studies of Secginli et al. [48], and Ghaffari et al. [49]. In a study done by Turkey et al. using the printed educational content, this score did not change [50]. This inconsistency might be due to different intervention methods and educational content. According to this model, women who believe to be susceptible to breast cancer and feel more at risk will more likely perform breast cancer screening behaviors. The present study used the incidence and mortality statistics of breast cancer in Iranian women and then the ones from Kashan in recent years to increase the perceived susceptibility.

Based on the research results, the educational intervention can increase the score of the perceived severity of the breast cancer in the intervention group compared to the control group. This result was consistent with the results of studies conducted by Ghaffari et al. [49], Ansarifar et al. [51], and Gözüm et al. [52]. The results indicate that if a person seriously understands the disease and its consequences, it will lead to preventive behaviors. In order to increase the perceived severity, the present study used medical images with involved organs of the actual patients as well as discussions about the complications which can affect the person gradually during the disease course. The perceived severity variable can also act as a double-edged sword, in other words, when the perceived severity is high, denial or failure to accept preventive behaviors may also occur.

The score of perceived barriers significantly decreased in the intervention group compared to the control group after the educational intervention in two periods of 3, and 6 months after the intervention. This finding is consistent with that of of Ghaffari et al. [49], Secginli et al. [48], and Park et al. [53]. When women have a better understanding of screening behaviors and reduce the barriers in performing such behaviors, the probability of performing these behaviors increases. The present study used the brain storming method to discuss the barriers against performing such behaviors and the ways to overcome such barriers according to the women’s point of view.

In terms of perceived benefits, there was a significant difference between the intervention and control groups. Their perceived benefits scores increased in the intervention group 3 and 6 months after the educational intervention. The result was consistent with the results of Shojaiezadeh et al. [54], Ghaffari et al. [49], and Secginli et al. [48]. In this study, lectures, questions and answers, and group discussion educational methods were used to expand the perceived benefits of conducting breast cancer screening methods.

The current study found that there was no significant difference between the two groups in terms of self-efficacy scores before the educational intervention; however, the scores of the intervention and control groups were significantly different after the intervention. This score increased in the intervention group due to the educational intervention. The results were consistent with the results of Aghamolaei et al. [55], Shojae Zadeh et al. [6], Sheykhan et al. [56], Sharoni et al. [57], studies in Malaysia [20] and Turkey [58]. Self-efficacy is the most important predictor of behavior change which reflects individuals’ confidence in their ability to perform the right behavior. To increase the self-efficacy of women in performing breast self-examination, slides and video clips were used to learn how to perform self-examination step by step. The practical demonstration and moulage were also used. The step-by-step demonstration of self-examination refers to the use of the educational method to divide behavior into smaller and more practical steps in order to increase self-efficacy in women.

The results of the study on subjective norms indicated that there was a significant difference between the intervention and control groups in scores of perceived subjective norms 3 and 6 months after the intervention, but the difference was not significant before the intervention. The results were consistent with the results of Khani Jeihooni et al. [59], Orabi et al. [60] and Sheppardet al. [61], but the “subjective norms” construct did not change in the intervention group after the educational intervention in a study by Sargazi et al. [44]. The “subjective norms” construct implies the influence of important people such as spouses, mothers, sisters, and friends on a person’s life and preventive behavior. In the present study, women in the intervention group were asked to watch the video clips with their families. To affect the husbands’ point of view, they were asked to participate in the attendance training session held by a non-communicable disease specialist, but unfortunately, it was not welcomed by them.

In regard to skills, the results revealed a significant difference in skill scores obtained by women in the intervention group compared to the control group. The scores increased in the intervention group 3 and 6 months after the intervention, while they decreased after the educational intervention in the control group. The result was consistent with studies of Costellia Talley et al. [62], Wood et al. [63] and Ghaffari et al. [49].

Furthermore, there was a significant difference between the intervention and control groups in terms of behavioral intention scores 3 and 6 months after the intervention. The result was consistent with studies of Khani Jeihooni et al. [59], Peyman et al. [64], Dezham et al. [65], and Bashirian et al. [66], but inconsistent with other studies [58, 67, 68]. The intention is a key factor in a person’s readiness to perform a behavior. Researchers believe that the more people intend to perform a behavior, the more likely they perform that behavior [34, 64].

The research results regarding performing three breast cancer screening behaviors, including monthly breast self-examination, clinical examination by the midwife or physician and mammography, indicated that the rate of performing such three behaviors increased significantly in the intervention group compared to the control group (Regarding the clinical examination by the midwife or physician, considering that this examination should be done routinely once a year, the results of the study showed a significant increase in this examination, which was due to the fact that a number of women in the intervention group after ultrasound or mammography were determined to have breast cysts, and these women were examined once or even twice by the midwife or physician at intervals of 3 and 6 months). This study produced results which corroborate the findings of a great deal of the previous work in mammography by Khani Jeihooni et al. [59], khalili et al. [6]; the findings are consistent with data obtained from monthly breast self-examination, Bashirian et al. [66], these results are in line with those of previous studies about monthly self- and clinical examination by Sheppard et al. [61] and Mohseni et al. [69]. However, the findings of the current study do not support the previous research given breast self-examination and mammography 2 months after the intervention [49]. The differences between the two groups after the educational intervention suggested the effectiveness of the educational intervention based on the Health Action Model (HAM).

### Study limitations

The present study had some limitations, the most important of which was educational intervention during the COVID-19 Pandemic which forced the researchers to use the non-attendance method in instructing the target group. Several attendance training sessions were also held as soon as the conditions were favorable enough.

It was also impossible to invite effective people (spouses, mothers, etc.) in performing screening behaviors by women due to the COVID-19 pandemy and time constraints.

Using self-report questionnaires was another limitation of the study. In identifying the environmental factors affecting the performance of breast cancer screening behaviors by women, it was not possible to plan for intervention at all levels because of time constraints.

Moreover, at the time of sampling for the study, due to the COVID-19 pandemy, the number of people who referred to comprehensive health service centers was too small and people just went to the centers to receive essential services such as children’s vaccinations or sometimes to receive midwifery services. It was practically impossible to reach the desired sample size using other sampling methods. For this reason, the convenience sampling method was used.

Another limitation of this study was the significant difference between the two intervention and control groups in terms of the occupation variable, which despite dividing the samples into two groups randomly, the number of housewives in the control group was more than the intervention group and the number of employed people in the intervention group was more than the control group.

### Study strengths

The strengths of the present study were as follows: The previous studies did not utilize this model to improve these three screening behaviors; hence, the innovation of the present study was that it used this model to develop educational content and target the educational intervention. Women in the target group had no personal or family history of breast cancer.

Furthermore, the present study used the opinions of the specialists in the non-communicable diseases unit of the Kashan health deputy about breast cancer and the status of its screening behaviors, as well as the opinions of specialists in the field of education and health promotion. Identifying the determinants of screening behaviors based on the opinions of the target group can create a suitable and effective framework for the implementation of a client-centered program.

## Conclusion

The findings clearly indicate that the educational intervention based on the Health Action Model was able to increase the scores of the constructs of this model and can also improve screening behaviors in the intervention group compared to the control group. The results showed that the educational intervention was able to make women more sensitive about the possibility of their disease, improve a greater understanding of the severity of the complications and consequences of breast cancer and motivate them to perform breast cancer screening behaviors. It also improved women’s behavioral intention to perform these behaviors and increased the self-efficacy and skills of women in performing monthly breast self-examination or attending clinical examination and mammography. Additionally, it could attract their support and accompaniment of women in performing these behaviors.

So, considering that the educational intervention in this study was conducted face-to-face and favorable results were obtained, and since the Health Action Model has several structures that can affect individual and social factors, it is suggested that the staff and practitioners in the field Health should use the structures of this model as a comprehensive framework for face-to-face educational interventions in comprehensive health service centers.

## Data Availability

The datasets used in this study are available from the corresponding author on request.

## Abbreviations

HAM: Health Action Model
PASS: Power and Sample Size
SPSS: Statistical Package for the Social Sciences

## References

[1] Bray F (2018). Global cancer statistics 2018: GLOBOCAN estimates of incidence and mortality worldwide for 36 cancers in 185 countries. CA Cancer J Clin.

[2] Zarif Yeganeh M, Toorang F, Ebrahimipour Koujan S. Nutrition and breast cancer: what do say meta-analyses. In: 5th Tehran breast cancer conference–abstract book. 2012.

[3] Parks R, Cheung K.-L. An overview of the Nottingham Research Programme on Primary Breast Cancer in Older Women: breast cancer in older women. Liaquat Med Res J. 2021;3(3):49–52.

[4] Bray F (2013). Global estimates of cancer prevalence for 27 sites in the adult population in 2008. Int J Cancer.

[5] Babu GR (2011). Breast cancer screening among females in Iran and recommendations for improved practice: a review. Asian Pac J Cancer Prev.

[6] Kalili S (2014). Effectiveness of training of health beliefs and attitude of women referred to Shahid Bahtash Clinic in Lavizan region, Tehran, regarding breast cancer screening methods using the model of health belief. Health J.

[7] Hoerger TJ (2011). Estimated effects of the national breast and cervical cancer early detection program on breast cancer mortality. Am J Prev Med.

[8] Ma F (2021). Interpretation of specification for breast cancer screening, early diagnosis, and treatment management in Chinese women. J Natl Cancer Center.

[9] Gencturk N (2013). The status of knowledge and practice of early diagnosis methods for breast cancer by women healthcare professionals. J Breast Health.

[10] Coleman C. Early detection and screening for breast cancer. In: Seminars in oncology nursing. WB Saunders; 2017;33(2):141–55. 10.1016/j.soncn.2017.02.009 28365057

[11] Miller BC. Cultural characteristics of young Black women as predictors of intentions to engage in potential future targeted recommendations for mammography screening and BRCA1/2 genetic testing (Doctoral dissertation, State University of New York at Stony Brook). 2020.

[12] Welch HG (2016). Breast-cancer tumor size, overdiagnosis, and mammography screening effectiveness. N Engl J Med.

[13] Asghari E (2016). The relationship between health belief and breast self-examination among Iranian university students. Int J Wom Health Reprod Sci.

[14] Brennan ME (2016). The role of clinical breast examination in cancer screening for women at average risk: a mini review. Maturitas.

[15] Noroozi A, Jomand T, Tahmasebi R (2011). Determinants of breast self-examination performance among Iranian women: an application of the health belief model. J Cancer Educ.

[16] Shiryazdi SM (2014). Health beliefs and breast cancer screening behaviors among Iranian female health workers. Asian Pac J Cancer Prev.

[17] Gupta A, Shridhar K, Dhillon P (2015). A review of breast cancer awareness among women in India: cancer literate or awareness deficit?. Eur J Cancer.

[18] Naghibi A (2016). Identification of factors associated with breast cancer screening based on the PEN-3 model among female school teachers in Kermanshah. Iran J Health Educ Health Promot.

[19] Kommula A, Borra S, Kommula VM (2014). Awareness and practice of breast self examination among women in south India. Int J Curr Microbiol App Sci.

[20] Akhtari-Zavare M (2015). Barriers to breast self examination practice among Malaysian female students: a cross sectional study. Springerplus.

[21] Baron-Epel O (2010). Attitudes and beliefs associated with mammography in a multiethnic population in Israel. Health Educ Behav.

[22] Abu-Helalah MA (2015). Knowledge, barriers and attitudes towards breast cancer mammography screening in Jordan. Asian Pac J Cancer Prev.

[23] Kissal A (2018). The effect of women’s breast cancer fear and social support perceptions on the process of participating in screening. Glob Health Promot.

[24] Fayanju OM (2014). Perceived barriers to mammography among underserved women in a Breast Health Center Outreach Program. Am J Surg.

[25] Abuidris DO (2013). Breast-cancer screening with trained volunteers in a rural area of Sudan: a pilot study. Lancet Oncol.

[26] Tones K, et al. Health promotion: planning & strategies. Health Promot. 2019;5019146:1–704.

[27] Vahedian-Shahroodi M (2019). Applying a health action model to predict and improve healthy behaviors in coal miners. Glob Health Promot.

[28] Mazaheri MA, Heidarnia A (2015). The effect of intervention based on health action model to promote workers’ safe behavior in Isfahan Steel Company. J Educ Health Promot.

[29] Nieto-Montenegro S, Brown JL, LaBorde LF (2008). Development and assessment of pilot food safety educational materials and training strategies for Hispanic workers in the mushroom industry using the Health Action Model. Food Control.

[30] John EJ, Obono ON, Michael IG, Iyamba EE. Food handling/serving and hygiene practices: the perception of food vendors operating in Obubra local government area of Cross river state, Nigeria. Int J Food Sci Nutr. 2018;3(6):250–6.

[31] NCSS L. PASS 15 power analysis and sample size software Kaysville. Utah; 2017. [Google Scholar].

[32] Mahboobighazaani F (2022). Design and psychometric evaluation of the breast cancer screening behaviors scale based on the health action model (HAM). BMC Womens Health.

[33] Khodayarian M (2019). Development and psychometric evaluation of a protection motivation theory–based scale assessing the adherence of Iranian women breast cancer prevention behaviors. Iran Q J Breast Dis.

[34] Rezabeigi-Davarani E (2016). Breast self-examination and its effective factors based on the theory of planned behavior among women in Kerman, Iran. J Educ Community Health.

[35] Ghahremani L (2016). Self-care education programs based on a trans-theoretical model in women referring to health centers: breast self-examination behavior in Iran. Asian Pac J Cancer Prev.

[36] Matlabi M (2018). The impact of educational intervention based on theory of planned behavior in breast self-examination of women referred to health centers. Horiz Med Sci.

[37] Dewi TK (2019). Determinants of breast self-examination practice among women in Surabaya, Indonesia: an application of the health belief model. BMC Public Health.

[38] Moodi M (2019). Predictors of breast self-examination behavior in housewives based on trans-theoretical model. J Birjand Univ Med Sci.

[39] Roberto A (2017). Personalised informed choice on evidence and controversy on mammography screening: study protocol for a randomized controlled trial. BMC Cancer.

[40] Ohuchi N (2016). Sensitivity and specificity of mammography and adjunctive ultrasonography to screen for breast cancer in the Japan Strategic Anti-cancer Randomized Trial (J-START): a randomised controlled trial. Lancet.

[41] Borrayo EA, Rosales M, Gonzalez P (2017). Entertainment-education narrative versus nonnarrative interventions to educate and motivate Latinas to engage in mammography screening. Health Educ Behav.

[42] Bakhtariagdam F, Nourizadeh R, Sahebi L (2012). The role of health belief model in promotion of beliefs and behaviors of breast cancer screening in women referring to health care centers of Tabriz in 2010. Med J Tabriz Univ Med Sci.

[43] Alameer A (2019). Effect of health education on female teachers’ knowledge and practices regarding early breast cancer detection and screening in the Jazan Area: a quasi-experimental study. J Cancer Educ.

[44] Sargazi M (2014). Eeffect educational intervention based on the theory of planned behavior leads to early detection of breast cancer in women referred to health centers in Zahedan. J Breast Dis Iran.

[45] Saatsaz S, et al. Effect of educational intervention on condition of knowledge and practice about breast cancer screening among employed teachers. 2010.

[46] Fazel N, et al. Breast self-examination: knowledge, and performance among upper 20 year old women in medical-health centers in sabzevar-Iran in 2010. 2010.

[47] Yılmaz M, Sayın Y, Cengiz HÖ (2017). The effects of training on knowledge and beliefs about breast cancer and early diagnosis methods among women. Eur J Breast Health.

[48] Secginli S, Nahcivan NO (2011). The effectiveness of a nurse-delivered breast health promotion program on breast cancer screening behaviours in non-adherent Turkish women: a randomized controlled trial. Int J Nurs Stud.

[49] Ghaffari M (2019). Evaluation of health belief model-based intervention on breast cancer screening behaviors among health volunteers. J Cancer Educ.

[50] Gursoy AA (2009). Comparison of three educational interventions on breast self-examination knowledge and health beliefs. Asian Pac J Cancer Prev.

[51] Ansarifar T (2000). Evaluating the Effect of Education based on the Health Belief Model in taking the preventive behaviors for breast cancer among female health workers.

[52] Gözüm S (2010). Effectiveness of peer education for breast cancer screening and health beliefs in eastern Turkey. Cancer Nurs.

[53] Park S (2009). Effects of a cognition-oriented breast self-examination intervention for Korean women and their spouses. Public Health Nurs.

[54] Khalili S, et al. The effectiveness of education on the health beliefs and practices related to breast cancer screening among women referred to Shahid Behtash Clinic, Lavizan area, Tehran, using health belief model. 2014.

[55] Aghamolaei T, et al. Improving breast self‐examination: an educational intervention based on health belief model. Int J Cancer Manag. 2011;4(2):e80735.

[56] Sheykhan R, Sepahvandi M, Ghazanfari F (2019). Changing attitude, subjective norm, perceived behavioral control, mammography self-efficacy regarding breast cancer screening: the effect of an educational intervention in women aged 40 to 60 years. J Woman Fam Stud.

[57] Sharoni SKA (2017). A self-efficacy education programme on foot self-care behaviour among older patients with diabetes in a public long-term care institution, Malaysia: a Quasi-experimental Pilot Study. BMJ Open.

[58] Tuzcu A, Bahar Z, Gözüm S (2016). Effects of interventions based on health behavior models on breast cancer screening behaviors of migrant women in Turkey. Cancer Nurs.

[59] KhaniJeihooni A, Darvishi N, Harsini PA (2020). The effect of educational intervention based on the theory of planned behavior on mammography screening in Iranian women. J Cancer Educ.

[60] Orabi E (2017). Effect of health education intervention on knowledge, and attitude regarding menopausal period among premenopausal female employees. Egypt J Community Med.

[61] Sheppard VB (2013). Promoting mammography adherence in underserved women: the telephone coaching adherence study. Contemp Clin Trials.

[62] Talley CH, Yang L, Williams KP (2017). Breast cancer screening paved with good intentions: application of the information–motivation–behavioral skills model to racial/ethnic minority women. J Immigr Minor Health.

[63] Wood RY (1996). Breast self-examination proficiency in older women: measuring the efficacy of video self-instruction kits. Cancer Nurs.

[64] Peyman N, Amani M, Esmaili H (2016). The relationship between health literacy and constructs of theory of planned behavior and breast cancer screening tests performance among women referred to health care centers in Roshtkhar, 2015. Iran Q J Breast Dis.

[65] Dezham S, Roozbahani N, Khorsandi M. Application of theory of planned behavior in predicting screening mammography in housewives over 40 years. 2015.

[66] Bashirian S (2021). Evaluation of an intervention program for promoting breast self-examination behavior in employed women in Iran. Breast Cancer Basic Clin Res.

[67] Heydari E, Noroozi A (2015). Comparison of two different educational methods for teachers’ mammography based on the Health Belief Model. Asian Pac J Cancer Prev.

[68] Baghianimoghadam MH (2011). The effect of education based on protection–motivation theory on skin cancer preventive practices among female high school students in Yazd. Horiz Med Sci.

[69] Mohsenipouya H (2019). Use of the health education campaign (HEC) in the field of breast cancer screening in the north of Iran. Health Educ Health Promot.

